# When masculinity interferes with women's treatment of HIV infection: a qualitative study about adherence to antiretroviral therapy in Zimbabwe

**DOI:** 10.1186/1758-2652-14-29

**Published:** 2011-06-09

**Authors:** Morten Skovdal, Catherine Campbell, Constance Nyamukapa, Simon Gregson

**Affiliations:** 1 London School of Economics and Political Sciences, UK; 2 Biomedical Research and Training Institute, Zimbabwe and Imperial College London, UK

## Abstract

**Background:**

Social constructions of masculinity have been shown to serve as an obstacle to men's access and adherence to antiretroviral therapies (ART). In the light of women's relative lack of power in many aspects of interpersonal relationships with men in many African settings, our objective is to explore how male denial of HIV/AIDS impacts on their female partners' ability to access and adhere to ART.

**Methods:**

We conducted a qualitative case study involving thematic analysis of 37 individual interviews and five focus groups with a total of 53 male and female antiretroviral drug users and 25 healthcare providers in rural eastern Zimbabwe.

**Results:**

Rooted in hegemonic notions of masculinity, men saw HIV/AIDS as a threat to their manhood and dignity and exhibited a profound fear of the disease. In the process of denying and avoiding their association with AIDS, many men undermine their wives' efforts to access and adhere to ART. Many women felt unable to disclose their HIV status to their husbands, forcing them to take their medication in secret, and act without a supportive treatment partner, which is widely accepted to be vitally important for adherence success. Some husbands, when discovering that their wives are on ART, deny them permission to take the drugs, or indeed steal the drugs for their own treatment. Men's avoidance of HIV also leave many HIV-positive women feeling vulnerable to re-infection as their husbands, in an attempt to demonstrate their manhood, are believed to continue engaging in HIV-risky behaviours.

**Conclusions:**

Hegemonic notions of masculinity can interfere with women's adherence to ART. It is important that those concerned with promoting effective treatment services recognise the gender and household dynamics that may prevent some women from successfully adhering to ART, and explore ways to work with both women and men to identify couples-based strategies to increase adherence to ART

## Background

Antiretroviral programmes are expanding throughout sub-Saharan Africa, providing people living with HIV and AIDS (PLHIV) with glimpses of hope [1]. However, antiretroviral therapy (ART) is complex, and treatment regimens must be carefully adhered to in order to avoid drug resistance [2] and improve survival [3]. This requires consistent and meticulous monitoring [4,5], and is most likely to be achieved with the support of a treatment partner [6-8], family members [8,9] and peers from the community [7,10]. The likelihood of successful ART adherence is optimized in contexts where there can be a certain openness and acceptance of their HIV status [1], allowing antiretroviral (ARV) users to negotiate support from significant others, and fit ART into their daily schedule, free from fear and stigma [11]. Disclosing HIV status and ART initiation to long-term partners are therefore often said to be key to ART adherence [12,13]. We report on a study of the ART user/service interface where men exhibited a profound fear of being associated with HIV/AIDS, and examine how gender constructs and couple relations influenced women's ability to disclose their status and adhere to ART in this setting.

In writing this paper, we are not seeking to represent men as controlling and women as passive victims. We fully acknowledge that more women than men take an active role in accessing HIV services [14,15], and women have been observed to respond more positively to the adherence of ART than men [16,17]. Such findings suggest that many women are able to take control of their health, more so than men. Nevertheless, while no other study has previously explored the link between masculinity and women's adherence to ART, an expanding number of studies have examined the negative impact of men's disengagement with HIV services to the uptake of antenatal voluntary counselling and testing (VCT) and mother to child transmission [18,19].

In Tanzania, for example, men's lack of participation in VCT was found to reduce the chances of their HIV-positive wives using nevirapine prophylaxis for the prevention of mother to child transmission and their chances of avoiding breastfeeding, as well as of adhering to alternative infant feeding methods [19]. However, a recent study suggests that if men are formally invited to attend antenatal care and couple's HIV testing, many men will attend with their wives, reducing mother to child transmission [20]. Understanding the processes that contribute to men's (dis)engagement with HIV services, and the impact on family members, is therefore key for the prevention and treatment of AIDS for family members [15].

The paper is framed within Connell's [21] concept of hegemonic masculinity, a social constructionist theory often used to explain differences in the health-seeking behaviours of men and women [22]. Connell argues that many societies subscribe to a dominant definition of masculinity that both reinforces and demonstrates male power in a given place and time and, in the process, subordinates women, as well as the other non-dominant forms of masculinity that will exist in any social setting, for example, homosexual masculinities or masculinities associated with less controlling attitudes to women. Hegemonic notions of "a real man" as tough, independent, physically strong, fearless and sexually unstoppable have been identified as key drivers of the AIDS epidemic in many African settings [23]. It is against this background, and in the light of our interest to draw attention to the impact of household dynamics on ART adherence, that we examine how masculinity and men's responses to HIV interfere with women's adherence to ART in rural Zimbabwe.

## Methods

The study, which forms part of an ongoing research project on factors shaping service access and treatment adherence in eastern Zimbabwe, was granted ethical approval from the Medical Research Council of Zimbabwe (A/681) and Imperial College London (ICREC_9_3_13). Written and informed consent was gathered from all research participants with the agreement that their identities would not be revealed. Pseudonyms have therefore been used throughout.

### Study setting and sampling

Participants for this qualitative study were recruited from three rural areas, where the majority of people sustain their livelihoods through subsistence farming or work on tea and coffee plantations. The rural areas are located in the Manicaland Province of Eastern Zimbabwe. The province is characterized by high levels of poverty and HIV, though the latter has seen some decline. The HIV prevalence rate stands at 18%, a decline from 23% over a five-year period [24]. In 2009, 215,000 people in Zimbabwe (57% of those living with AIDS) accessed ART [25]. These trends are testimony to the positive progress that has been made to curtail the epidemic and make ART accessible in Zimbabwe. This study draws on the perspectives of active ARV users and nurses, focusing on their understandings and retrospective experiences of factors shaping their access and adherence to ART. ARV users were independently sampled using a mix of snowball (using village community health workers), opportunistic (self-selected informants) and typical case (adherers to ART) sampling. None of the ARV users were husbands and wives. Nurses from three health facilities were recruited on the basis of their willingness to participate in the study.

### Data collection and analysis

The research methods used for this study were individual interviews and focus group discussions (FGDs). Both these methods allow researchers to explore areas of interest in depth, but as FGDs involve the interaction between a group of people, the methods may yield different information. As we wanted to explore the effect of masculinity on women's ART adherence, we were particularly keen on bringing women together in FGDs, hoping that this would reveal similarities and differences of experiences and that the sharing of experiences would enable them to articulate the nuances of their experiences. We conducted 19 individual interviews and four FGDs with a total of 32 female and 21 male ARV users, and 18 individual interviews and one FGD with a total of 11 female and 14 male health staff (see Table 1). Each FGD had an average of eight participants. Individual interviews averaged one hour and group interviews averaged 2.3 hours.

**Table 1:** Summary of participants and research methods

	Interviews	*Male*	*Female*	FGD	*Male*	*Female*	Participants	*Male*	*Female*
Nurse	**18**	9	9	**1**	5	2	**25**	14	11
Patient	**19**	13	6	**4**	8	26	**53**	21	32
Total	**37**	22	15	**5**	13	28	**78**	35	43

Interview topic guides were the same for both individual interviews and FGDs, and explored such areas as counselling and testing uptake and treatment adherence, disclosure of HIV and experiences at the healthcare centre. To gather more specific information about different experiences of HIV service access, participants were asked: "What factors do you think have the most impact on access?", "Why do some patients fail to present themselves at services?", "Can you give me an example of a person with HIV who was good at accessing HIV services?" and "Can you give me an example of a patient who failed to access the services at the best time?".

Most respondents referred to gender differences, and non-directive probing questions were used to encourage informants to expand on these. In addition to questions from the interview topic guide, ARV users participating in FGDs were invited to role play "a good visit to the health centre" and "a bad visit to the health centre"; this was to gain insight into the interaction of service users and providers in this context. The role plays allowed us to explore the characteristics of a typical service user/provider interaction, While the role playing involved only a few of the FGD participants (the role play lasted five to 10 minutes), everyone contributed to the planning of the role play and the discussion about it afterwards.

Interviews were conducted by three experienced Shona-speaking fieldworkers and were audio recorded, with permission from the informants. These were subsequently transcribed and translated into English by the fieldworkers and imported into Atlas.Ti, a qualitative analysis software package, for coding (providing text segments with descriptive headings). The coding was done by two people who coded the data independently from each other. The analysts subsequently discussed emerging themes. The coding process generated a total of 225 codes. We have reported on some of these codes elsewhere [1,7,26-28] and will make no attempt to discuss all of these codes in this paper. For this paper, we used Attride-Stirling's [29] thematic network analysis to help us identify codes and themes relevant to our interest in the impacts of masculinities on women's ART access and adherence (Table 2). This process generated 26 codes, covering 12 basic themes, which are discussed in our Results section. These basic themes were further clustered into three more inclusive "organising themes", which serve as the sub-headings for our findings, which follow.

**Table 2:** Coding framework: Pathways through which masculinity impacts on women's opportunities for adherence

Codes	Basic themes identified	Organizing themes
- Men feel superior - Strong and resilient - Independent and tough - Pride - Can't show fear - Men are heads of house - Men have girlfriends - Women not allowed extra marital relationships	1. Men are perceived as physically strong and capable of withstanding disease. 2. Men are perceived as emotionally independent and tough. 3. Men should not show fear. 4. Men are perceived as breadwinners and the ones to carry out heavy duties, while women work at home, providing care for children and supporting husbands.	Social constructions of masculinity
- Fear of being recognised as HIV positive - Having AIDS exposes their promiscuity - Embarrassment - Fear disclosing status to their wives - Fear being alone - HIV compromises their manhood - Denying it can happen to them - Death over dishonour - Blaming others - Not taking AIDS seriously - Avoiding talking about AIDS	5. Men are afraid of being recognised as HIV positive as it exposes their promiscuity and he may lose his dignity. 6. Men have guilt about sleeping around and feel so embarrassed that they often fear disclosing their status to their wives. 7. Married men fear being abandoned by their wives and young men fear being rejected by girls and living a life alone. 8. Many men deny that they are ill from AIDS and would rather blame others or die with "dignity".	Men's fear and denial of HIV
- Women fear disclosing HIV status to husbands - Men stop wives from taking drugs - Men's denial compromises women's treatment - Men steal women's tablets - Men's denial can re-infect women - Couples counselling needed - Using sex to demonstrate manhood	9. Men's lack of participation in HIV services, coupled with their sexuality, leaves women susceptible for re-infection. 10. Because of men's negative reactions to HIV, many women fear disclosing their status to their husbands, losing out on an important treatment partner. 11. Out of shame, men may deny their wives taking ART. 12. If husbands know that their wives are on ART and suspect they are sick too, they may steal their wives' ARVs.	Masculinity interfering with married women's ART adherence

## Results

### Social constructions of masculinity

To understand how masculinity interferes with women's ART adherence, this sub-section gives detail on how hegemonic notions of masculinity influence men's experiences of HIV. Both male and female nurses and ARV users made reference to hegemonic notions of masculinity, describing "a real man" as physically strong, tough and resilient to illness, independent, responsible and successful in sustaining his family. For a man to contract HIV and develop AIDS is therefore perceived as a threat to his sense of masculinity:

I really felt such HIV tests were going to embarrass me and make me feel useless. As a man I have that pride of being the father, the husband and head of the family and can you imagine an HIV positive result will just wash away all that respect. (Joseph, patient)

There is that pride and men feel that being ill is a women's issue because it is rather belittling to be seen coming to the hospital every now and then as it is a sign of weakness. (Philip, patient)

The idea that being ill belittles a man's sense of manhood and role as head of household and that hospitals are seen as female territories highlights the conflict that exists between socially constructed notions of masculinity and HIV.

### Men's fear and denial of HIV

To mediate the links between social constructions of masculinity and husbands' interference with their wives' ART adherence, we now turn to examining the conflict that exists between masculinity and HIV and how this has led many men in this context to develop a profound fear of any association with HIV, preventing them from getting tested and disclosing their HIV status:

There are people in this community who are not feeling well and know that they should go and get tested for HIV, yet they are afraid to ... The problem is very common with men, men are afraid to come out in the open and face the reality. (Henry, patient)

In giving detail to their fear of HIV and AIDS, male participants spoke about some of the consequences of testing HIV positive to their manhood. For example, men reported that that testing positive would represent them as promiscuous and irresponsible:

For me, it kind of gives people an impression that I have been sleeping around carelessly, which is not true at all. We have been given information that this disease mainly affects people who sleep around carelessly, and, when one tests HIV positive, it kind of confirms to the public that I have been having careless extra-marital sexual relationships. I was really worried, and I couldn't come to terms with an HIV positive result. (Liyod, patient)

Such representation, coupled with their HIV status, made married men fear that their wives would leave them, while younger men felt that women would have no interest in them and that they would remain bachelors for the rest of their lives:

If positive, some young men feel they would lose their chances of getting a woman to marry them. (Henry, patient)

Nurses and ARV users of both genders articulated the pressure that men are under to conform to hegemonic notions of masculinity and their fear of the potential consequences of being HIV positive. As illustrated by Henry, this can lead men to deny the fact that they may be HIV positive and delay seeking HIV testing and treatment. The men participating in this study spoke about their difficulties in coming to terms with their HIV status and admitted that it was only when they were very ill and had developed AIDS that they got tested and enrolled onto ART. Not only does the profound fear that is embedded in men's experience of HIV impact negatively on men's own health, it can also, as we will now discuss, interfere with women's treatment of HIV.

### Masculinity interfering with married women's ART adherence

While women in this context were good at making use of HIV services, they faced a number of obstacles in their efforts to adhere to ART, many of which related to their husbands' fear of association with HIV. Women who had gone to get tested and tested positive and knew of their husbands' fear of HIV were often afraid of telling their husbands about their HIV seroconversion:

I know a certain lady who attended these HIV/AIDS functions and decided to take up HIV tests and she tested HIV positive, but she could not tell her husband even though she suspected him of being the one who infected her. (Constantine, patient)

Women unable to share with their husbands their HIV status and their need to comply with a strict treatment programme miss out on getting support from family members, compromising their ability to adhere successfully.

Men's non-disclosure of their HIV status, as well as their position in deciding whether or not to use condoms when having sex with their wives, can leave women open for infection or re-infection, particularly if their husbands are not on ART [30]. A number of women on ART spoke about their husbands' denial of their HIV status and their continued promiscuity, fearing re-infections that might complicate their treatment regimen:

Men are a problem. Women on ART come to support group activities, yet their husbands do not come and we don't know whether these men have been tested. In these support groups, we are taught to have protected sex to avoid re-infection, but the husbands do not come. If I am on ART but my husband is still unwilling to get tested for HIV, he will refuse to have protected sex, yet I am already on ART and at risk of being re-infected. (Martha, patient)

A number of nurses, when speaking about challenges to women's adherence to ART, recognised the role of their clients' husbands and often felt demotivated by their efforts to support women on ART as their advice to female patients was often undermined by their patient's husbands:

I am very unsatisfied and I feel pulled down when I am dealing with a female patient whose male counterpart refuses to come for [an] HIV test. You see this means your efforts are in vain because you treat her and she goes to be re-infected at home because the husband disregards condom use. (Roselyn, nurse)

Rooted in men's fear of association with HIV, nurses frequently spoke about how husbands could prevent their wives from attending monthly review dates and picking up their antiretroviral drugs - interfering with women's adherence to ART. Some men were reported to have stolen their wives' hospital cards in an attempt to prevent them from going to the clinic:

Some women say their husbands deny them the right to come to the hospital saying "you want to expose me that I am HIV positive", so they even go further by stealing their wives' hospital cards. However, such husbands need counselling. (Tsitsi, nurse)

Another commonly reported strategy used by husbands to disassociate themselves with AIDS and prevent their wives from going to the hospital and adhering to their treatment was to threaten their wives with divorce if they continued to make use of HIV services:

Women when they come to get services from the opportunistic infection (OI) clinic and they are initiated on ART, the husband will then threaten to divorce the wife if she continues taking antiretroviral drugs. This will then affect her ability to adhere to ART. (Weston, nurse)

Finally, and reflecting men's own fear of enrolling onto an ART programme, husbands were reported to steal ARVs from their wives to take themselves:

... as you ask into why she did not adhere she will begin to open up and she may even cry telling you the real problem; "I have a problem, my husband doesn't want to come to OI clinic, when I get my monthly supply, he will grab my tablets and take them himself." (Weston, nurse)

In this subsection, we have outlined some of the many varied ways through which men's fear of HIV and AIDS, rooted in hegemonic notions of masculinity, can prevent their wives from adhering successfully to ART.

## Discussion

Through our discussion of social constructions of masculinity, men's fear and denial of HIV, and how such responses interfere with women's adherence to ART, we have presented an account of the dynamic relationship between hegemonic notions of masculinity and married women's adherence to ART. As men perceived being ill from HIV/AIDS as a threat to their manhood (e.g., men as strong, in control of their sexuality, resilient to illness and capable of being the breadwinners), men feared HIV/AIDS, a fear intensified by prospects of being represented as promiscuous or ending up without a life companion. As a result of this fear, men actively sought to disassociate themselves from any activities that might link them to HIV/AIDS. In this process, men presented a number of obstacles to their wives who felt more comfortable in accessing HIV services, interfering with their opportunities for optimal adherence to ART. They did so by increasing their risk of re-infection and preventing them from attending hospital appointments and from taking their medicines. In summary, our findings suggest that husbands' commitments to hegemonic notions of masculinity can prevent their wives from adhering to ART.

The relevance and generalizability of these findings deserve some discussion. As we spoke with ART users who reported retrospectively about their experiences in coming to terms with their HIV seroconversion and uptake and adherence to ART, we felt it was important to corroborate their views with those of nurses and other health staff, obtaining the views of health staff who dealt with cases of non-adherence at the time of this study. To move beyond individual accounts and experiences, we sought to map out the responses and meanings that our informants articulated, guided by our theoretical framework and research aim, in order to develop a narrative and interpretive understanding of the role of masculinity in interfering with women's adherence to ART. However, as this study was conducted in a unique context, we acknowledge that generalizability can only be achieved through the investigation of masculinity on women's ART adherence in other contexts.

Furthermore, as the study was exploratory, it did not seek to compare and contrast responses across different socio-demographic groups. Future research exploring the pathways between masculinity and its impact on women's adherence to ART should therefore consider the trajectory of discordant and HIV-infected couples, and their age and marital status, as well as the clinical characteristics of patients, Nevertheless, we believe that our findings provide a potential explanation for previous studies that have highlighted the negative impact that men's disengagement with HIV services can have on mother to children transmissions [18,19], adding to previous knowledge by illustrating the link between masculinity and men's disengagement with HIV services and how this impact family members.

## Conclusions

Certain recommendations can be made from our analysis. First, it makes little sense to HIV test and treat only one of the partners in a relationship. Although couple testing and ART enrolment has had positive outcomes in some settings, couple-focused programmes are still not widespread [31]. Second, there is an urgent need for HIV services to consider the gender and household dynamics that prevent men from making use of the services and for women to successfully adhere to treatment. They can do this either by providing opportunities for men to deconstruct hegemonic notions of masculinity and create spaces where masculinities can be renegotiated and transformed *or *by creating therapeutic environments that are friendlier and aligned to local masculinities. As masculinities are negotiated and constructed at a community level, we believe that ART programmes need to scale up community-outreach programmes that consult local men about their fears of HIV/AIDS and develop responses accordingly.

Writing in South Africa, Colvin and Robins [32] have found local social support groups for men particularly effective in creating such spaces. Also in South Africa, the Men as Partners (MAP) programme by the EngenderHealth organization and the Planned Parenthood Association has found systematic discussions of masculinity and the involvement of men to be key in addressing the gender issues, power dynamics and gender stereotypes that contribute to women's marginalized position in responding to HIV/AIDS [33,34]. Bila and Egrot [15] recently concluded from their study on gender asymmetry to healthcare facility attendance among PLHIV in Burkina Faso that to reduce women's vulnerability and strengthen their responses to ART adherence, one must understand men's disengagement with HIV services.

We have in this paper, and elsewhere [35], moved this debate forward and outlined the relationship that exists between hegemonic notions of masculinity, men's disengagement with HIV services and couple-based obstacles that women face in ART adherence.

## Competing interests

The authors declare that they have no competing interests.

## Authors' contributions

MS managed the data set, conducted the data analysis, and wrote the first draft of the article. CC supervised the data collection and analysis; and contributed to the final version of the article. CN managed and conducted the fieldwork. SG was the principal investigator of the overall Manicaland Project, which hosted this research. All authors were involved in the write-up and approved the final submission of the article and its contents.
